# Performance of the Xpert HIV-1 Viral Load Assay: a Systematic Review and Meta-analysis

**DOI:** 10.1128/JCM.01673-17

**Published:** 2018-03-26

**Authors:** Madlen Nash, Sophie Huddart, Sayema Badar, Shrikala Baliga, Kavitha Saravu, Madhukar Pai

**Affiliations:** a Department of Epidemiology, Biostatistics and Occupational Health, McGill University, Montreal, Canada; b McGill International TB Centre, McGill University, Montreal, Canada; c Department of Epidemiology, Tulane University School of Public Health and Tropical Medicine, Tulane, New Orleans, Louisiana, USA; d Department of Microbiology, Kasturba Medical College, Mangalore, Manipal Academy of Higher Education, Manipal, India; e Manipal McGill Center for Infectious Diseases, Manipal Academy of Higher Education, Manipal, India; f Department of Medicine, Kasturba Medical College, Manipal, Manipal Academy of Higher Education, Manipal, India; Memorial Sloan Kettering Cancer Center

**Keywords:** HIV, Xpert HIV-1, accuracy, diagnosis, viral load

## Abstract

Viral load (VL) is the preferred treatment-monitoring approach for HIV-positive patients. However, more rapid, near-patient, and low-complexity assays are needed to scale up VL testing. The Xpert HIV-1 VL assay (Cepheid, Sunnyvale, CA) is a new, automated molecular test, and it can leverage the GeneXpert systems that are being used widely for tuberculosis diagnosis. We systematically reviewed the evidence on the performance of this new tool in comparison to established reference standards. A total of 12 articles (13 studies) in which HIV patient VLs were compared between Xpert HIV VL assay and a reference standard VL assay were identified. Study quality was generally high, but substantial variability was observed in the number and type of agreement measures reported. Correlation coefficients between Xpert and reference assays were high, with a pooled Pearson correlation (*n* = 8) of 0.94 (95% confidence interval [CI], 0.89, 0.97) and Spearman correlation (*n* = 3) of 0.96 (95% CI, 0.86, 0.99). Bland-Altman metrics (*n* = 11) all were within 0.35 log copies/ml of perfect agreement. Overall, Xpert HIV-1 VL performed well compared to current reference tests. The minimal training and infrastructure requirements for the Xpert HIV-1 VL assay make it attractive for use in resource-constrained settings, where point-of-care VL testing is most needed.

## INTRODUCTION

Despite the recommendations put forward by the World Health Organization (WHO), viral load (VL) monitoring of antiretroviral therapy (ART) is not routinely performed in many low-resource countries, and treatment failure is diagnosed on the basis of clinical or immunological criteria. Currently used VL assays demand sophisticated facilities, expensive equipment, and skilled technicians, making them unaffordable and largely impractical for scale-up in resource-limited settings (1). To expand the use of targeted and routine VL monitoring, inexpensive, low-complexity assays are needed, preferably for point-of-care use.

The Xpert HIV-1 VL assay (Cepheid, Sunnyvale, CA, USA), performed on the GeneXpert instrument system, is an *in vitro* diagnostic test designed for the rapid quantification of HIV-1 in human plasma from individuals with an active HIV infection. It uses real-time quantitative reverse transcription PCR technology and targets HIV-1 group M subtypes A, B, C, D, AE, F, G, H, AB, AG, J, and K and groups N and O. It has a limit of quantitation of 40 copies/ml and can detect HIV-1 RNA over a linear range of 40 to 10,000,000 copies/ml. The GeneXpert platform allows on-demand molecular testing in one fully integrated closed cartridge and provides results in 90 min (2). The Xpert HIV-1 VL assay runs on the same GeneXpert platform as the WHO-endorsed Xpert MTB/RIF cartridge, used for diagnosis of tuberculosis (TB).

The Xpert MTB/RIF assay is a major advance in tuberculosis diagnostics, and more than 23 million cartridges have been used around the world (3, 4). Given the high coprevalence of TB and HIV in many settings (5) and the need for greater integration of TB-HIV services, leveraging the existing GeneXpert network for HIV may substantially increase access to VL testing. Similar to the Xpert MTB/RIF assay, the Xpert HIV-1 VL assay demands minimal training and modest infrastructure requirements while providing rapid results. If proven to be as accurate as current, established reference standard VL tests, this monitoring tool has the potential for rapid scale-up in countries already using the GeneXpert platform for TB. Scaling up routine HIV-1 VL testing is essential to meet the UNAIDS 90-90-90 targets (6).

In July of 2017, the Xpert HIV-1 VL was accepted for the WHO list of prequalified *in vitro* diagnostics (7). Country-level validation studies on the accuracy of this assay have now been conducted in a variety of settings. We conducted a systematic review to synthesize evidence from these validation studies and estimate the overall agreement between the Xpert HIV-1 VL assay and current reference standard assays.

## MATERIALS AND METHODS

We conducted a systematic review of the literature according to PRISMA guidelines (8). A written protocol for the systematic review was prepared *a priori*. A modification to the pooling criteria was made after the authors were made aware of the tendency of *I*^2^, a measure of heterogeneity among studies, to approach 100% as sample sizes of studies increase (9). Thus, for the pooling of correlations, where internal study variability was very small and the resulting *I*^2^ was very high, the decision to pool was based on clinical heterogeneity.

### Search strategy and selection criteria.

Using Ovid, we systematically searched Medline (1946 to 2017), Embase (1947 to 2017), and Global Health (1973 to 2017) for studies evaluating the performance of the Xpert HIV-1 VL assay in HIV-positive patients (see Appendix S1 in the supplemental material for complete search strategy). No language or geographic restriction was applied. The study search was conducted on 25 September 2017.

All studies that enrolled participants with a known HIV-positive status were eligible for inclusion.

**FIG 1:**
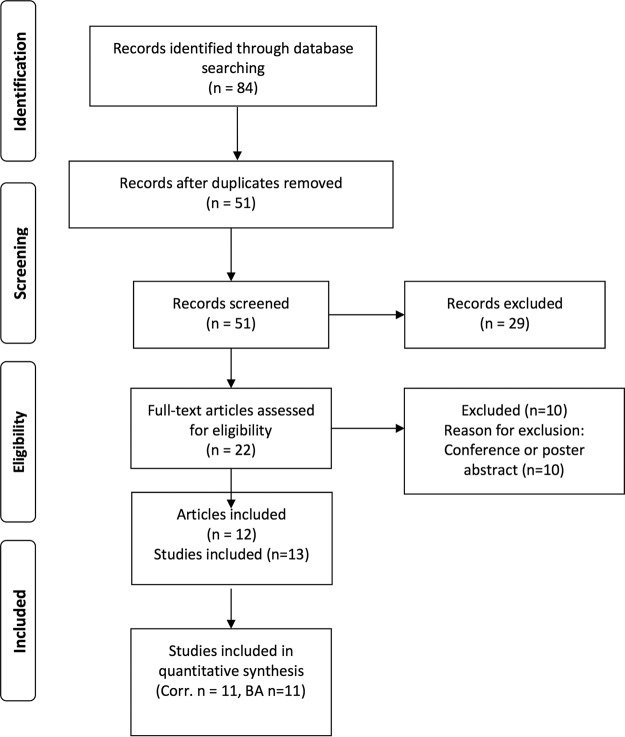
PRISMA flow chart.

We restricted inclusion to studies of patients of known HIV status, because VL testing is not recommended as an HIV diagnostic but as a treatment-monitoring tool. The included studies were required to compare Cepheid's Xpert HIV-1 VL assay to another established PCR-based VL assay and report at least one comparison measure between Xpert VLs and gold standard VLs (i.e., Pearson or Spearman correlation, Bland-Altman agreement, etc.). Conference abstracts and posters were excluded, as their quality could not be comprehensively assessed (Fig. 1).

Two reviewers (S. Huddart and M. Nash) screened the titles and abstracts of citations retrieved from all sources. Duplicates were removed, and studies which met the inclusion criteria were flagged for further review. A full-text screen of relevant studies was then performed by the same independent reviewers.

### Data extraction.

Two reviewers (S. Huddart and M. Nash) independently extracted data from all eligible studies on patient demographics, correlation coefficients, and results of Bland-Altman analyses comparing Xpert HIV-1 VL assays and the reference test and study quality (see Appendix S1).

Extracted data were adjudicated by the two reviewers, with discrepancies resolved by a third reviewer (S. Badar). Study authors were contacted regarding information not reported in the included manuscripts, but no responses were received.

### Risk of bias and assessment of study quality.

A modified version of the QUADAS-2 criteria for diagnostic tests was used to assess study quality (10). Because of the numerical output of both the index and reference tests, biased interpretation of either assay was unlikely; thus, we did not evaluate blinding of the index test or reference test as sources of bias. We assessed patient selection and patient flow according to QUADAS-2 guidelines. Overall risk of bias for patient selection and flow was summarized as low, high, or unclear.

### Statistical analysis.

Correlation coefficients, both Spearman and Pearson, were extracted from studies reporting these measures. Because of the theoretical heterogeneity between parametric and nonparametric measures, meta-analysis was only considered within strata of correlation type. The decision to pool was based on the authors' assessment of clinical heterogeneity.

The results of Bland-Altman analyses, a summary of the mean differences between VL measures, were also extracted. Twelve Bland-Altman analyses were performed among the included studies, but one (11) did not report the corresponding standard deviation, confidence interval (CI), or limit of agreement. Without a measure of variance, it is difficult to appropriately interpret this Bland-Altman analysis, thus the study's Bland-Altman analysis was not included in the quantitative summary. The decision to pool for Bland-Altman was based on *I*^2^, as internal study variability was moderate and *I*^2^ appropriately reflected heterogeneity.

All extracted studies performed their correlation and Bland-Altman analyses on log-transformed viral loads. For studies on patient samples, samples were independent, with one sample per patient included in the agreement analyses. For one included study using laboratory-quality assessment samples, the sampling procedure was not described (reference 12, referring to the laboratory samples).

Meta-analysis was performed using a DerSimonian and Laird random effects model (13).

The number of studies was insufficient to conduct metaregression. Traditional methods to assess publication bias, such as Egger's test or funnel plots, are severely underpowered, especially with small numbers of studies, thus publication bias was not formally assessed but is assumed to exist to some degree in all systematic reviews.

All statistical analyses were performed in R.

## RESULTS

Figure 1 shows the study selection flow chart. Twelve articles covering 13 studies were identified during the systematic search of the literature; these studies enrolled more than 3,300 individual patients, 2,011 of whom had VLs quantifiable on Xpert and the reference test (Table 1) (11, 12, 14–23). For most studies, patient blood samples were collected as part of routine clinical practice either within a larger HIV/AIDS study or through health care institutions. One paper included data on the quality assessment samples (12). Three studies enrolled HIV patients in India (20, 22, 23), and three studies enrolled HIV patients in South Africa (references 12 [referring to both the patient and the laboratory samples] and 14). Overall, the quality of reporting of patient demographics was poor. Only 3 of 13 studies reported a complete set of demographic covariates (average patient age, gender distribution, and the proportion of participants receiving ART) (12, 14, 20). Of those studies that reported ART coverage, treatment rates ranged from 0 to 100%. The reference standards used included PCR-based VL assays (Abbott RealTime HIV-1 m2000rt, Abbott Molecular; COBAS AmpliPrep/COBAS TaqMan, Roche Diagnostics; NucliSENS EasyQ HIV-1 v2.0, bioMérieux; and Versant HIV-1 RNA 1.5 assay, Siemens HealthCare Diagnostics).

**TABLE 1:** Study characteristics

Study	Yr	No. enrolled	No. quantifiable on index and reference test	Location	Patient population	Age	% Female	% on ART	Test and manufacturer^*a*^
Avidor et al. (21)	2017	383	254	Israel	HIV^+^, routine care				Roche TaqMan
Bruzzone et al. (19)	2017	50	41	Italy	HIV^+^ laboratory samples				Versant
Ceffa et al. (11)	2015	300	274	Malawi	Pediatric and adult HIV^+^, routine care			100	Abbott
Garrett et al. (14)	2016	42	42	South Africa	HIV^+^ women in CAPRISA 002 study	33	100	66	Roche TaqMan
Gous et al., laboratory samples (12)	2016	42	20	South Africa	Quality assessment HIV^+^ laboratory samples				Roche TaqMan
Gous et al., patient samples (12)	2016	158	53	South Africa	HIV^+^, routine care	42	54	100	Roche TaqMan
Gueudin et al. (15)	2016	295	162	France	HIV^+^, routine care			20	Abbott
Jordan et al. (16)	2016	764	390	Europe, USA	Adult HIV^+^, routine care	45	28		Abbott
Kulkarni et al. (22)	2017	219	167	India	HIV^+^, routine care	38		52	Abbott
Mor et al. (17)	2015	404	146	Israel	HIV^+^, routine care				NucliSens v2.0
Moyo et al. (18)	2016	302	277	Botswana	HIV^+^, ART naive, rural patients			0	Abbott
Nash et al. (20)	2017	246	89	India	Adult HIV^+^, routine care	41	40	70	Roche TaqMan
Swathirajan et al. (23)	2017	103	96	India	HIV^+^, routine care				Abbott

a Full names and manufacturers of reference tests are the following: Abbot, Abbott RealTime HIV-1 m2000rt, Abbott Molecular; Roche TaqMan, COBAS AmpliPrep/COBAS TaqMan, Roche Diagnostics; NucliSENS, NucliSENS EasyQ HIV-1 v2.0, bioMérieux; Versant, HIV-1 RNA 1.5 assay, Siemens HealthCare Diagnostics.

Overall, when pertinent information was reported, study quality was high (Table 2). Validation studies require cross-sectional design, which minimizes the risk of potential biases. Risk of selection bias, nongeneralizability, or risk of flow bias was generally low. Some studies did not clearly state aspects related to study design (i.e., sequential or random enrollment and reasons for exclusion from analysis), which limits the ability to draw conclusions about study quality.

**TABLE 2:** Study quality assessment using modified QUADAS-2^*a*^

Study	Risk of selection bias	Risk of nongeneralizability	Risk of flow bias
Avidor et al. (21)	Unclear	Unclear	Low
Bruzzone et al. (19)	Unclear	Low	Low
Ceffa et al. (11)	Low	Low	Low
Garrett et al. (14)	Low	Low	Low
Gous et al. (12)	Unclear	Unclear	Unclear
Gous et al. (12)	Unclear	Low	Unclear
Gueudin et al. (15)	Unclear	Low	Low
Jordan et al. (16)	Low	Low	High
Kulkarni et al. (22)	Unclear	Unclear	Low
Mor et al. (17)	Unclear	Unclear	Low
Moyo et al. (18)	Unclear	Unclear	Unclear
Nash et al. (20)	Low	Low	Low
Swathirajan et al. (23)	Unclear	Unclear	Low

a Risk of biases assessed using modified QUADAS-2 (10). Unclear risk denotes there was insufficient information provided in the paper to assess the particular bias.

**FIG 2:**
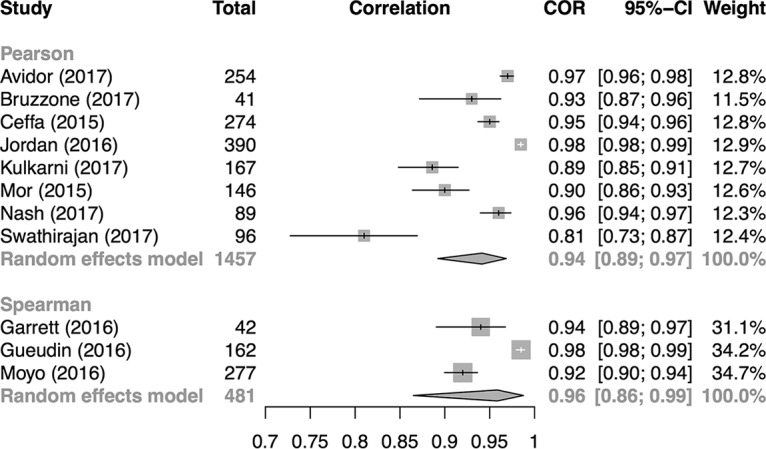
Forest plot for Pearson and Spearman correlation coefficients from comparison of VL values by Xpert and a reference test for VL.

All reported correlations between the Xpert HIV-1 VL assay and gold standard tests were very high for both Pearson and Spearman correlation coefficients (Fig. 2). As all correlations within strata were very close, ranging from 0.81 to 0.98, studies were pooled within strata of correlation type. The pooled correlations were 0.94 (95% CI, 0.89, 0.97) and 0.96 (95% CI, 0.86, 0.99) for Pearson and Spearman correlations, respectively. These values indicate a very high degree of agreement between Xpert VL and reference standard VL values.

**FIG 3:**
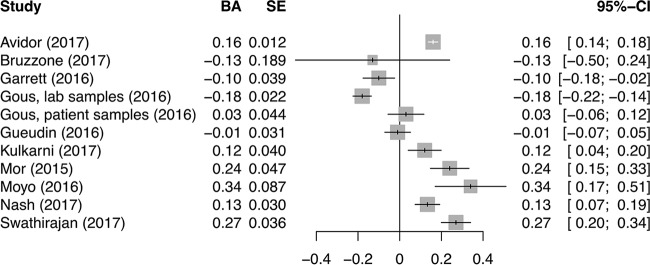
Forest plot for Bland-Altman (BA) correlation coefficients from comparison of VL values by Xpert and a reference test for VL.

Bland-Altman results are normally distributed, thus I^2^ is an appropriate metric for heterogeneity. The *I*^2^ for the Bland-Altman results was 96%, which suggests enough heterogeneity to preclude pooling. However, all studies reported a mean difference within 0.34 U of zero (the ideal value) (Fig. 3). We stratified the correlation and Bland-Altman values by ART status and found no major difference in studies with patients on ART versus studies with ART-naive or mixed-ART-status patients (see Fig. S1 and S2 in the supplemental material).

There was a sufficient number of Bland-Altman studies to stratify by type of reference test, although heterogeneity remained too high to provide pooled estimates (Fig. S3). No major differences in Bland-Altman results by reference test were identified.

We observed substantial variability in the number and type of agreement measures reported in the included studies (Table 3). Correlation, either Pearson or Spearman, and Bland-Altman measures were the most commonly reported, but several other measures were reported. Additionally, one study (11) reported a mean log difference as part of its Bland-Altman analysis but did not provide standard deviations, confidence intervals, or limits of agreement. This prevented some studies from being included in the Bland-Altman meta-analysis.

**TABLE 3:** Diversity of measures of agreement reported in Xpert HIV evaluations

Measure	Result by study^*a*^												
	Avidor et al. (21)	Bruzzone et al. (19)	Ceffa et al. (11)	Garrett et al. (14)	Gous et al., laboratory samples (12)	Gous et al., patient samples (12)	Gueudin et al. (15)	Jordan et al. (16)	Kulkarni et al. (22)	Mor et al. (17)	Moyo et al. (18)	Nash et al. (20)	Swathirajan et al. (23)
Pearson correlation											*		
Spearman correlation													
Bland-Altman			**					***					
Passing Bablok regression													
Deming regression													
Agreement, binary threshold (%)													
Agreement, 3+ categories (%)													
Similarity (%)													
Similarity coefficient of variation (%)													
Kappa Statistic													
Concordance Correlation													

a *, Study estimated Pearson correlation as a secondary metric, so the Spearman correlation was used for this analysis; **, study performed a Bland-Altman analysis but did not report a measure of variance and thus was excluded from the relevant analyses; ***, study provided a Bland-Altman plot but did not report the numerical mean log difference, which we deemed necessary to be classified as a Bland-Altman analysis. Shaded cells indicate that the measure was reported in the cited study. Blank cells indicate that the measure was not reported.

## DISCUSSION

Overall, our systematic review showed Xpert HIV-1 VL performs well compared with current established reference standard VL assays, both when measured by correlation and by Bland-Altman analysis. These findings might help inform policy guidance on this new assay, along with data on costs, feasibility, clinical impact, and cost-effectiveness.

Our systematic review has several potential limitations. First, as the search was conducted shortly after Xpert HIV-1 VL received WHO *in vitro* diagnostic prequalification, few validation studies had been conducted and published at the time of the search. Consequently, only 12 articles meeting our inclusion criteria were identified and included. We hope to update our meta-analysis in the future to account for new studies that will emerge on the Xpert VL assay. Second, the decision to exclude conference abstracts resulted in the exclusion of multiple relevant studies. Abstracts were excluded because study quality could not be effectively assessed, but as Xpert HIV-1 VL is a new tool, many studies on the assay have yet to be published as full-length articles. However, due to poor reporting, study quality was challenging to assess even in full-length articles. Where sufficient information was available, quality was high.

The final limitation pertains to generalizability. Many studies failed to report important demographic and clinical information, such as gender distribution, age of study participants, and patient ART status. This information is critical to understand the clinical relevance and generalizability of the reported data. Authors of studies with missing information were contacted; however, no responses were received. There was also substantial variability in which measures of agreement were calculated. To improve the ability to systematically review and pool the literature, a minimum set of standard measures of agreement should be agreed upon and used. Correlation and Bland-Altman could provide such a minimum set, as they measure both general agreement and magnitude and direction of bias between assays. Many studies reported percent agreement analyses where Xpert HIV-1 VL and the reference test were used to classify patients above or below certain thresholds. However, the specific thresholds used varied considerably, preventing a meaningful comparison. The use of a standard clinical threshold in future evaluations of Xpert HIV-1 VL would allow for direct comparisons in systematic reviews. The generalizability of our study is also limited due to the geographical distribution of the primary studies included; they were exclusively performed in countries where the predominant HIV subtype is C (e.g., India and Africa) or B (e.g., Israel, Europe, and the United States) (24). As more validation studies continue to be published on the Xpert assay, the results of this meta-analysis can be updated to include data from different countries where other HIV-1 subtypes predominate.

The purpose of this study was to assess VL agreement between Xpert and other gold standard assays as a measure of accuracy. However, a final recommendation for the use of Xpert VL will need to consider not just accuracy but also country-specific implementation research that addresses the feasibility of Xpert VL and its impact on patient outcomes.

The low-complexity nature of the Xpert HIV-1 VL assay, coupled with its ability to deliver same-day test results, make this technology particularly well suited for use in resource-constrained settings, where point-of-care VL testing is most needed (6). Deployment of such a tool, as well as utilization of preexisting Gene Xpert systems that are used in TB diagnostics, has the potential to increase access to VL testing, which will be necessary to achieve the 90-90-90 global targets for HIV/AIDS. Further research is needed to assess the impact of this VL assay on important patient outcomes and to establish its cost-effectiveness compared to VL assays currently used in centralized laboratories.

## Supplementary Material

Supplemental material
