# Supplementary material for: Performance of the Xpert HIV-1 Viral Load Assay: a Systematic Review and Meta-analysis
Source: J Clin Microbiol. 2018 Mar 26;56(4):e01673-17. doi: 10.1128/JCM.01673-17 (PMC5869835; doi:10.1128/JCM.01673-17)
Supplement: Supplemental material [file supp_56_4_e01673-17__index.html]

Performance of the Xpert HIV-1 Viral Load Assay: a Systematic Review and Meta-analysis — Supplemental material 

# Performance of the Xpert HIV-1 Viral Load Assay: a Systematic Review and Meta-analysis

## Supplemental material

- Supplemental file 1 -

  Appendix S1 and Fig. S1 (Forest plot for Pearson and Spearman correlation coefficients from comparison of VL values by Xpert and a reference test for VL stratified by patient ART status), S2 (Forest plot for Bland-Altman correlation coefficients from comparison of VL values by Xpert and a reference test for VL stratified by patient ART status), and S3 (Forest plot for Bland-Altman correlation coefficients from comparison of VL values by Xpert and a reference test for VL stratified by reference test)

  PDF, 751K
