# Supplementary material for: Performance of the Xpert HIV-1 Viral Load Assay: a Systematic Review and Meta-analysis
Source: J Clin Microbiol. 2018 Mar 26;56(4):e01673-17. doi: 10.1128/JCM.01673-17 (PMC5869835; doi:10.1128/JCM.01673-17)
Supplement: Supplemental material [file JCM.01673-17_zjm999095894s1.pdf]

***Appendix***

*Search strategy:*

Medline: (exp HIV-1/ OR HIV-1.mp.) AND (Xpert.mp. OR GeneXpert.mp. OR Cepheid.mp.)

Embase: (exp Human immunodeficiency virus 1/ OR HIV-1.mp.) AND (Xpert.mp. OR GeneXpert.mp. OR Cepheid.mp.)

Global Health: (HIV-1.mp.) AND (Xpert.mp. OR GeneXpert.mp. OR Cepheid.mp.)

*Extracted variables:*

First author

Study year

Location (country of experiment, if multiple will record all)

Source population

Avg. patient age

Percentage female

Percentage on ART

Sample size (# samples tested by both reference and index test)

Index test

Proportion patients w/ valid index test results

Reference test

Proportion patients w/ valid reference test results

Proportion of reference quantifiable samples that were quantifiable with index

Did any included participants not receive the reference test

Did any included participants not receive the index test

Were any patients excluded from the analysis

Proportion samples with invalid Xpert HIV-1 viral load results

Reasons for exclusion

Type of correlation

Number of samples used for correlation (ie quantifiable results)

Correlation on viral load

Correlation CI lower level

Correlation CI upper level

Correlation p value

Number of samples used for BA analysis correlation

Bland-Altman threshold

Bland-Altman agreement

Bland-Altman lower limit of 95% agreement

Bland-Altman upper limit of 95% agreement

Other method reported

Other method mean

Other method SD/confidence interval

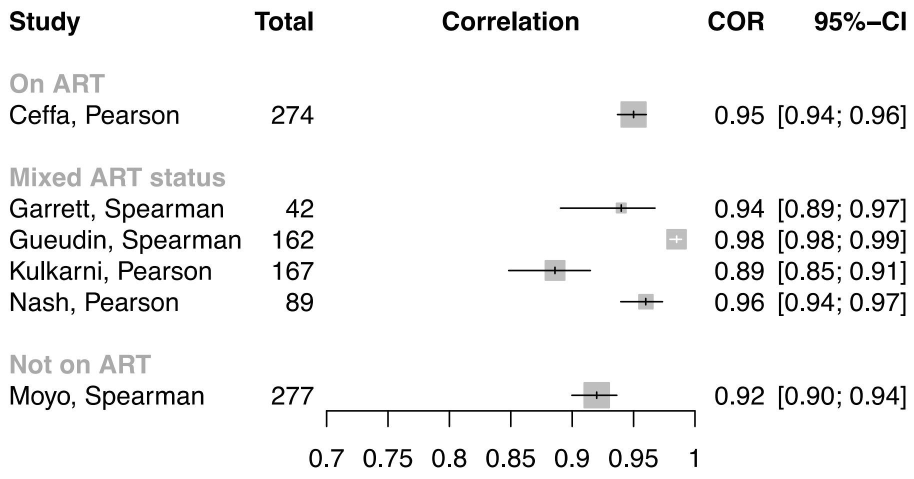


**Figure S1.** Forest plot for Pearson and Spearman correlation coefficients from comparison of VL values by Xpert and a reference test for VL stratified by patient ART status. Studies where ART status was not reported were excluded.

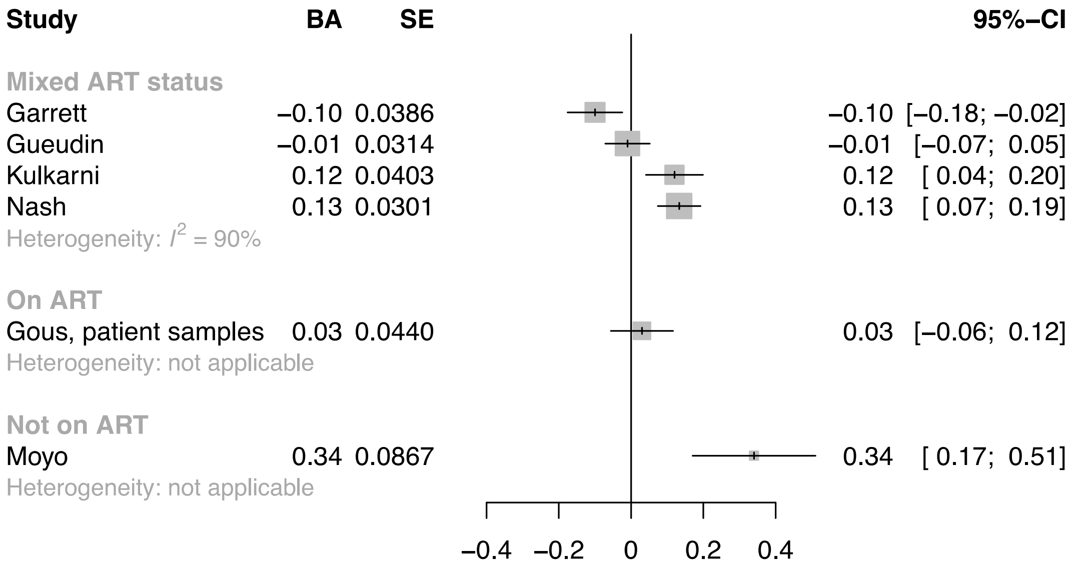


**Figure S2**. Forest plot for Bland-Altman (BA) correlation coefficients from comparison of VL values by Xpert and a reference test for VL stratified by patient ART status. Studies where ART status was not reported were excluded.

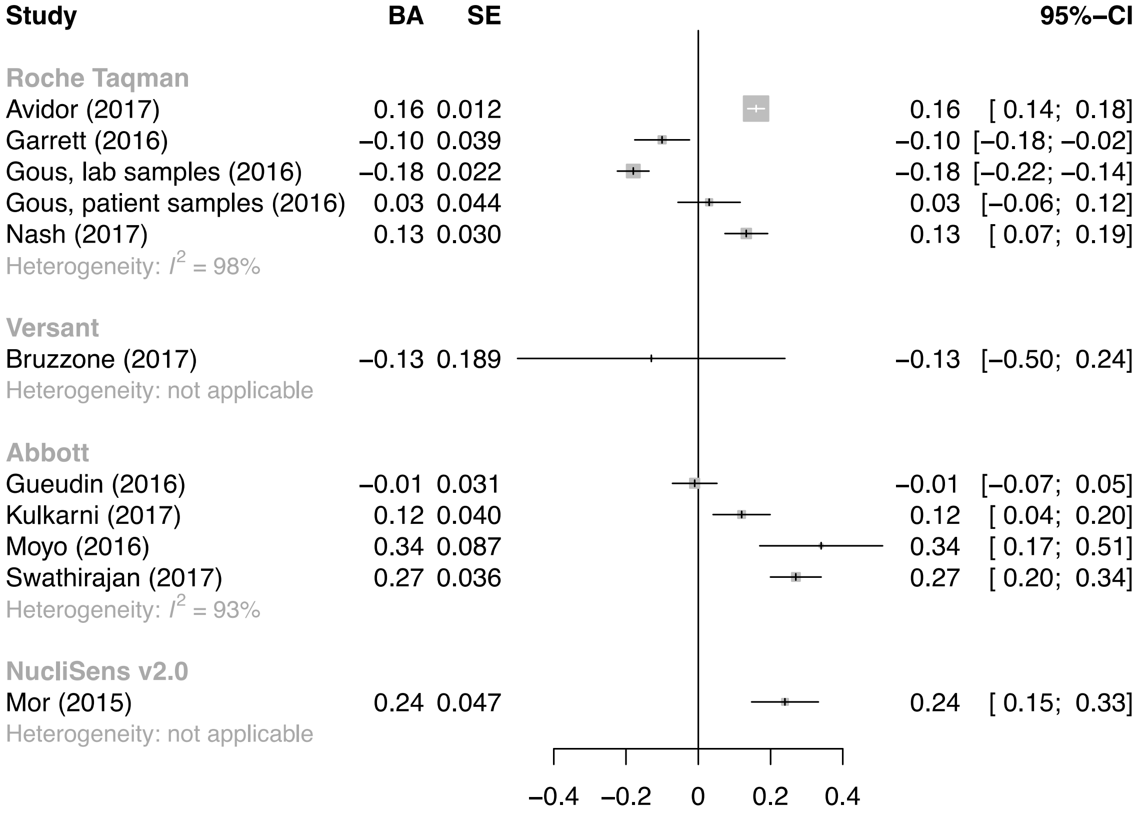


**Figure S3**. Forest plot for Bland-Altman (BA) correlation coefficients from comparison of VL values by Xpert and a reference test for VL stratified by reference test. Full names and manufacturers of reference tests are as follows: Abbot = Abbott RealTime HIV-1 m2000rt, Abbott Molecular. Roche Taqman = COBAS® AmpliPrep / COBAS® TaqMan®, Roche Diagnostics. Versant = NucliSENS EasyQ® HIV-1 v2.0, bioMérieux, VERSANT HIV-1 RNA 1.5 Assay, Siemens HealthCare Diagnostics
